# A Remembrance of Mary Ellen Avery, M.D.

**DOI:** 10.3389/fped.2014.00019

**Published:** 2014-04-22

**Authors:** Julie R. Ingelfinger

**Affiliations:** ^1^Pediatric Nephrology, MassGeneral Hospital for Children, Massachusetts General Hospital, Boston, MA, USA; ^2^Department of Pediatrics, Harvard Medical School, Boston, MA, USA

**Keywords:** mentors, mentoring, pediatrics, leadership, commentary

I was a recently hired member of the newly established Division of Pediatric Nephrology and an Instructor in Pediatrics at Harvard Medical School when Mel Avery became the Chief of the Department of Medicine and Physician-in-Chief at Children’s in 1974. There was excitement, as Dr. Avery’s appointment was a “first” unfolding right in front of those of us who, as young women physicians, hoped to have careers in academic pediatrics, and anticipatory talk filled the corridors of Children’s. On her arrival, Mel Avery was an automatic role model for the women among the faculty. She had made a major scientific discovery that a lack of surfactant caused respiratory distress syndrome in neonates, and she was still young (in her mid-forties), with abundance of energy. I was delighted and proud to have a woman, and one so accomplished, become the Physician-in-Chief at Children’s. Her hiring and her achievements allowed us to see that a woman could have a prominent role in academic medicine. The sense I had as a young faculty member was that Dr. Avery expected hard work and dedication, which was a self-selected trait among those women in the Department of Pediatrics at Children’s. So, the fit for us was a good one.

It was a boost in the arm to see that Dr. Avery supported women in medicine, and she was instrumental in asking Dr. Mary Ellen Wohl, who had come to Children’s in 1969, to become the chief of the Division of Pediatric Respiratory Diseases, and in the ensuing years Dr. Wohl became a pioneer in her own right. And in 1975, Dr. Lynne Reid, a pulmonary pathologist became the Chief of Pathology at Children’s.

I feel it is important while thinking about Mel Avery to remember that at the time she went to medical school at Johns Hopkins, few women were entering the field medicine – not all schools accepted women, and those that did still, accepted few per class. There were 4 in her class (of 90). An extraordinary student, she had been encouraged by a next-door neighbor who was a physician, the pediatrician Dr. Emily Bacon. Mel’s trajectory was admirable, in that she made her seminal discovery at a relatively young age. The number of women in most classes had more than doubled by the time I went to medical school, but it was still pitifully low. And Dr. Avery predicted that before long at least a third of most medical school classes would be women – a prediction that anticipated an increase but falls short of current statistics.

I do not think Mel fully understood how important a mentor she was for those of us who were house officers and junior faculty, even those of us with whom she did not work closely as individuals. What she shared in formal interviews, for example, with Georgia Litwack, with whom she subsequently wrote a book, would have had an extremely empowering effect had she been able to share it more directly with those of us who were coming along in the department at that time. However, a number of people became closer to her over time, with excellent effect.

Dr. Avery realized that the expectations of patients and the public were changing, and she made efforts that Children’s would pay attention to this trend, which proved prescient. I think she helped Children’s get ready for these changes.

In some published interviews, Mel acknowledged those things in which she was and was not comfortable. She was most at home with younger, not older children, and she always wanted to do her best. She noted that cutting corners made her tense. Mel appeared to be somewhat uncomfortable in many situations, and she was not a natural politician – probably not surprising given the era and her wish to complete all charges and tasks to perfection.

I thought she was also, in a certain way, shy. What I perceived as Mel Avery’s shyness and reticence to speak of her accomplishments was, in my view, a mixed blessing. It was refreshing to have a chief who did not seem to have a big ego, as many do. She clearly had vision, but it was hard to ferret it out on a daily basis. But vision, she had, and it was something I particularly appreciated.

One of her lasting accomplishments while at Children’s was the establishment of the Joint Program in Neonatology at Children’s and the other nearby Harvard Hospitals. It strengthened the residency program and was an improvement for all the programs concerned. Under Mel’s hand, neonatology became a major strength in Boston Pediatrics.

After her years as Physician-in-Chief at Children’s, Mel turned her attention to global health, and was particularly concerned about human rights and socioeconomic disparities. She worked hard with UNICEF as a health ambassador, advocating world wide to encourage polio vaccination and oral rehydration therapy.

While thinking about Mel Avery, I feel that her contributions were both focused and broad. I feel fortunate to have had her as my chief.

## Conflict of Interest Statement

The author declares that the research was conducted in the absence of any commercial or financial relationships that could be construed as a potential conflict of interest.

